# Effects of irrigation rates on cotton yield as affected by soil physical properties and topography in the southern high plains

**DOI:** 10.1371/journal.pone.0258496

**Published:** 2021-10-26

**Authors:** Jasmine Neupane, Wenxuan Guo, Charles P. West, Fangyuan Zhang, Zhe Lin

**Affiliations:** 1 Department of Plant and Soil Science, Texas Tech University, Lubbock, TX, United States of America; 2 Department of Soil and Crop Sciences, Texas A&M AgriLife Research, Lubbock, TX, United States of America; 3 Department of Mathematics and Statistics, Texas Tech University, Lubbock, TX, United States of America; Soil and Water Resources Institute ELGO-DIMITRA, GREECE

## Abstract

Lack of precipitation and groundwater for irrigation limits crop production in semi-arid regions, such as the Southern High Plains (SHP). Advanced technologies, such as variable rate irrigation (VRI), can conserve water and improve water use efficiency for sustainable agriculture. However, the adoption of VRI is hindered by the lack of on-farm research focusing on the feasibility of VRI. The objective of this study was to assess the effect of irrigation rates on cotton yield as affected by soil physical properties and topography in the Southern High Plains. This study was conducted in two fields within a 194-ha commercially managed farm in Hale County, Texas, in 2017. An irrigation treatment with three rates was implemented in a randomized complete block design with two replications as separate blocks in each field. A total of 230 composite soil samples were collected from the farm in spring 2017 and analyzed for texture. Information on apparent soil electrical conductivity (EC_a_), elevation, and final yield were collected from the fields. A statistical model showed that the effect of irrigation rates on cotton yield depended on its interaction with soil physical properties and topography. For example, areas with slope >2% and sand content >50% had no significant response to higher irrigation rates. This model suggests that applying irrigation amounts based on the yield response can be a basis for VRI. This study provides valuable information for site-specific irrigation to optimize crop production in fields with significant variability in soil physical properties and topography.

## Introduction

The Southern High Plains (SHP), located in the southern part of the Great Plains region of the United States, has a semi-arid climate with average annual precipitation ranging from 260 to 600 mm. The median summer rainfall (292 mm) is about one-third of the potential crop evapotranspiration (846 mm), so producers irrigate the fields to maximize economic returns [1]. The primary source for irrigation is the Ogallala Aquifer, which has been depleting rapidly in recent years with a negligible recharge rate [2]. Agriculture is considered the main cause for this shortage of water [3], as about 95% of groundwater pumped is for irrigation [4]. Currently, about 60% of the total cultivated area in the SHP is irrigated, with cotton as the dominant crop in the SHP. In 2016, half of the Texas cotton production area was concentrated in the SHP region [5]. Although growing cotton demands less water compared to corn (*Zea mays* L.) and other crops [6], in the present situation, the scarcity of freshwater is posing a threat to cotton production in the SHP. Hence, there is a need for decision tools to optimize the use of irrigation water [7]. Dryland farming is an option, but irrigation consistently maximizes net returns [8].

Recent research suggests the site-specific application of water, or variable rate irrigation (VRI), can be a solution to reducing water use and improving water use efficiency (WUE) [9]. VRI is a technology that applies varying water rates to match spatial variability in soil and plant characteristics based on water needs rather than applying a uniform rate throughout the field [10]. VRI consists of several components such as the pivot irrigation system, solenoid valves, control nodes, and sprinkler head nozzles which can be manipulated for variable application of water [11]. The state-of-the-art of VRI provides farmers with individual nozzle control, which makes it highly effective in reducing water use and increasing WUE of crops by applying the right amount of water at the right place and at the right time [12]. A review of VRI across different crop and weather regimes of the world concluded that VRI could save 10–15% of irrigation water [11] and increase crop productivity compared to uniform application. Another study reported a more than 20% increase in crop water productivity for corn and sorghum [*Sorghum bicolor* (L.) Moench] using VRI [13].

However, the feasibility and effectiveness of VRI in the commercial fields depend on the size of the field, crop species, weather, and physicochemical field properties [14–16]. Although the VRT technology, such as variable rate irrigation (VRI), is relatively mature, the decision support systems are not well established or reliable enough for the farmer to use directly in their field [15]. Several studies have shown that the challenges in the adoption of VRT technology are lack of skill to process and utilize the data, the scale of agriculture (field scale vs plot scale), and most importantly, lack of scientifically validated procedures that determine the variable rate application of inputs and their profitability [17, 18]. Most of the studies for VRI have been conducted in small-scale experimental fields that do not consider the large-scale variability present in the field and, therefore, are not justified for use in the real world situation [19]. Comprehensive studies at the field scale are needed to understand the factors contributing to the spatial yield variability as affected by irrigation rates. An experiment conducted in Tennessee found that a uniform level of irrigation did not result in uniform lint yield in all parts of the field owing to differences in soil texture and available water holding capacity. Similarly, yield varied significantly across years in the same part of the field having the same irrigation level indicating the importance of soil properties, topography, and weather conditions in crop production [20]. Hence, understanding how the intrinsic properties of the field influence yield at a given level of input is primarily important as a baseline for VRI application in any field.

The hypothesis is that the incorporation of spatial variation in topography and soil properties related to water management can improve water savings by optimizing water applications at different landscape positions. The goal of this study was to develop strategies to optimize irrigation for enhanced production, using cotton as an indicator crop in a semi-arid region. The specific objective was to assess the effect of irrigation rates on cotton yield as affected by soil physical properties and topography in the Southern High Plains. This objective was achieved by assessing the cotton yield and field properties in two fields within a 194-ha commercial farm with a center-pivot VRI system.

## Materials and methods

### Experimental site and design

**Fig 1:**
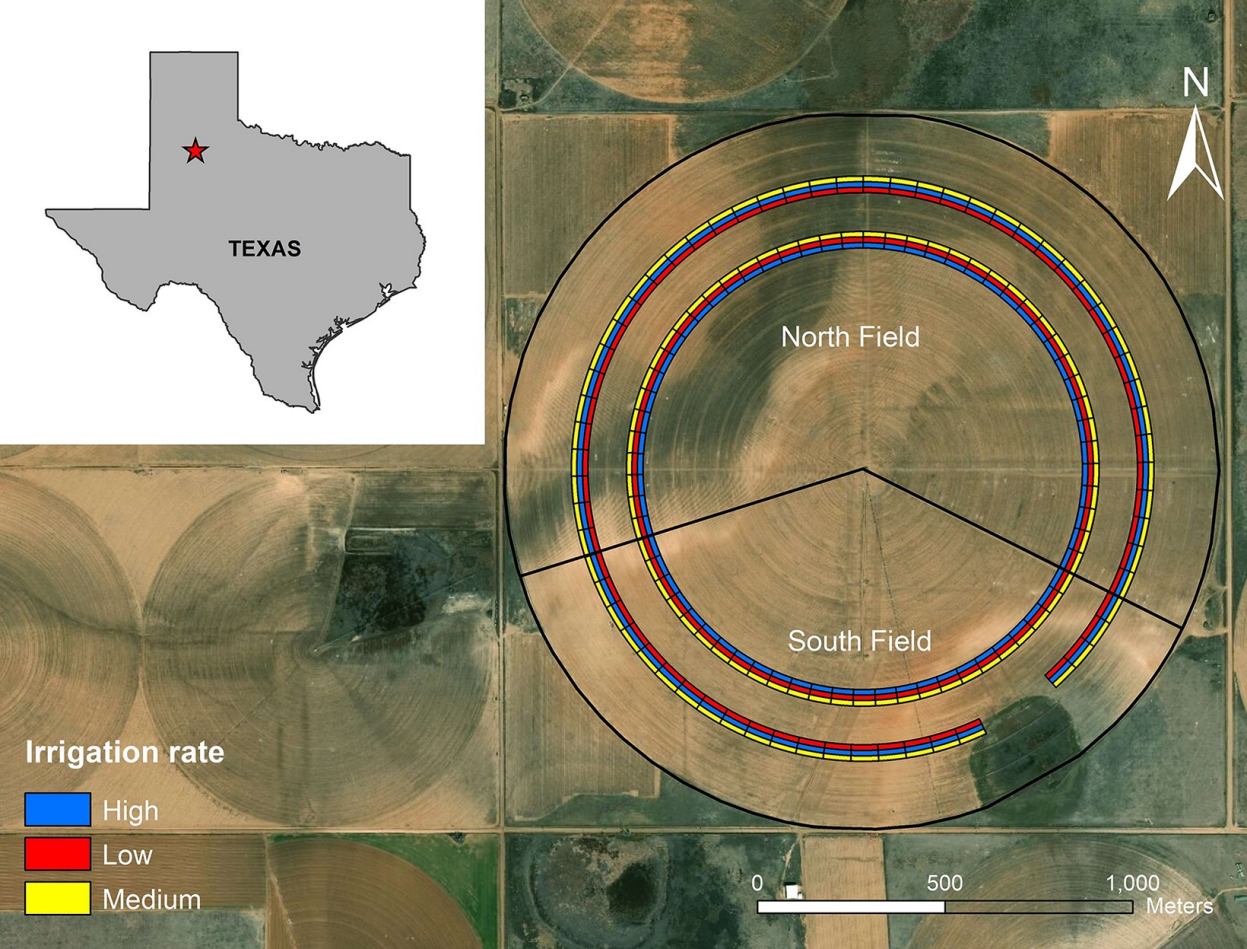
Study site with irrigation treatment design in two fields of a 194-ha farm in Hale County, Texas in 2017 (basemap source: Esri, DigitalGlobe, GeoEye, i-cubed, USDA FSA, USGS, AEX, Getmapping, Aerogrid, IGN, IGP, swisstopo, and the GIS User Community).

This experiment was conducted in two fields, North Field (122 ha) and South Field (72 ha), within a 194-ha farm in Hale County, Texas, in 2017 (Fig 1). Hale County is located in the southern portion of the Ogallala Aquifer. This semi-arid area has average rainfall ranging from 260 mm to 600 mm per year. The median summer rainfall (292 mm) is about one-third of the potential crop evapotranspiration of 846 mm [1]. The research farm is located in the northwest part of Texas (33°57’26.31" N, 101°47’20.31" W). The farm has an uncultivated playa lake on the south side. The experimental design in each field consisted of a randomized complete block design with three irrigation depths–high (25.4 mm), medium (12.7 mm), and low (0 mm) applied in approximately 13 days cycle of center pivot system. Each treatment was replicated twice, resulting in six 8-row strips across the fields. Furthermore, each strip was divided into 66 plots, each 50 m long and 12 m wide. The North Field had 246 plots, and the South field had 141 plots in total. The ArcGIS (Version 10.5, ESRI, Redlands, CA) was used to create a shapefile consisting of the treatment polygons as a prescription map. In the North Field, the cotton cultivar FiberMax (FM 2011GT, BASF, Ludwigshafen, Germany) was planted in 1-m row spacing on May 20, 2017, at the rate of 14,400 seeds ha^-1^. In the South Field, the cotton cultivar DP 1522 B2XF (Deltapine Monsanto Company, St. Louis, MO) was planted at the rate of 12,000 seeds ha^-1^ on the same day. These two fields were managed together across the boundary, such as planting, fertilization, irrigation, and harvesting.

#### Irrigation system

**Fig 2:**
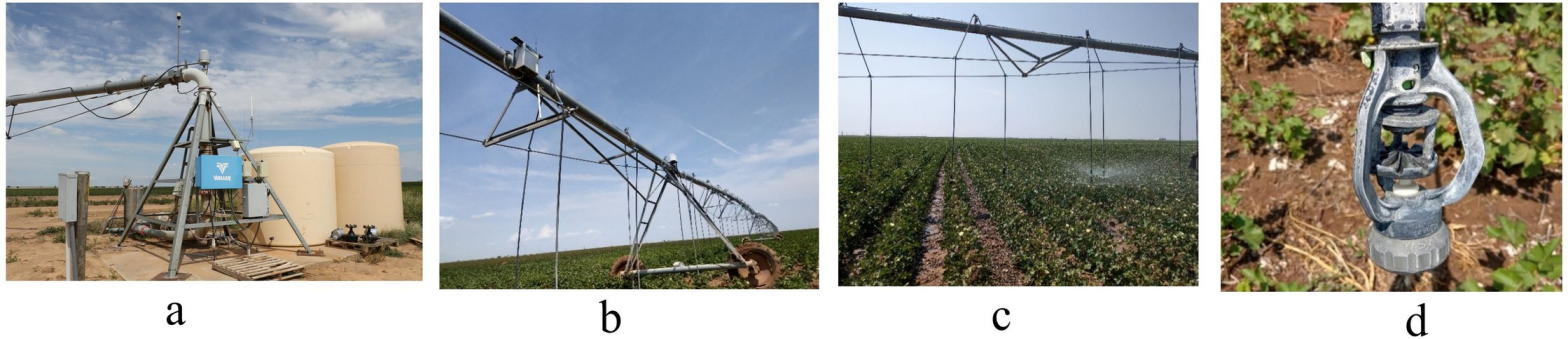
Components of a variable rate irrigation system in a 194-ha farm in Hale County, Texas (a. Control panel, b. Center pivot, c. Variable application of water, and d. Sprinkler head).

Irrigation was applied using a Valley 8000 standard pivot irrigation system coupled with the Trimble Irrigate-IQ VRI zone control package. It was 780 m long and covered this 194-ha farm with 18 spans. Each experimental plot consisted of an equal number of sprinklers applying three irrigation rates throughout the field. The Valley VRI controller included the zone control unit, solenoid valves, a GPS receiver, and software. The zone control unit controlled the duty cycle of the sprinklers by turning electric solenoid valves on and off to achieve desired application depths in individual control zones. VRI prescriptions were created using the software provided with the VRI system using the Trimble Irrigate-IQ. Fig 2 shows the components of the VRI present in the field. Pre-season irrigation was applied between March 18^th^ and planting (May 20^th^); In-season irrigation was applied between planting and September 9^th^.

#### Weather data

**Fig 3:**
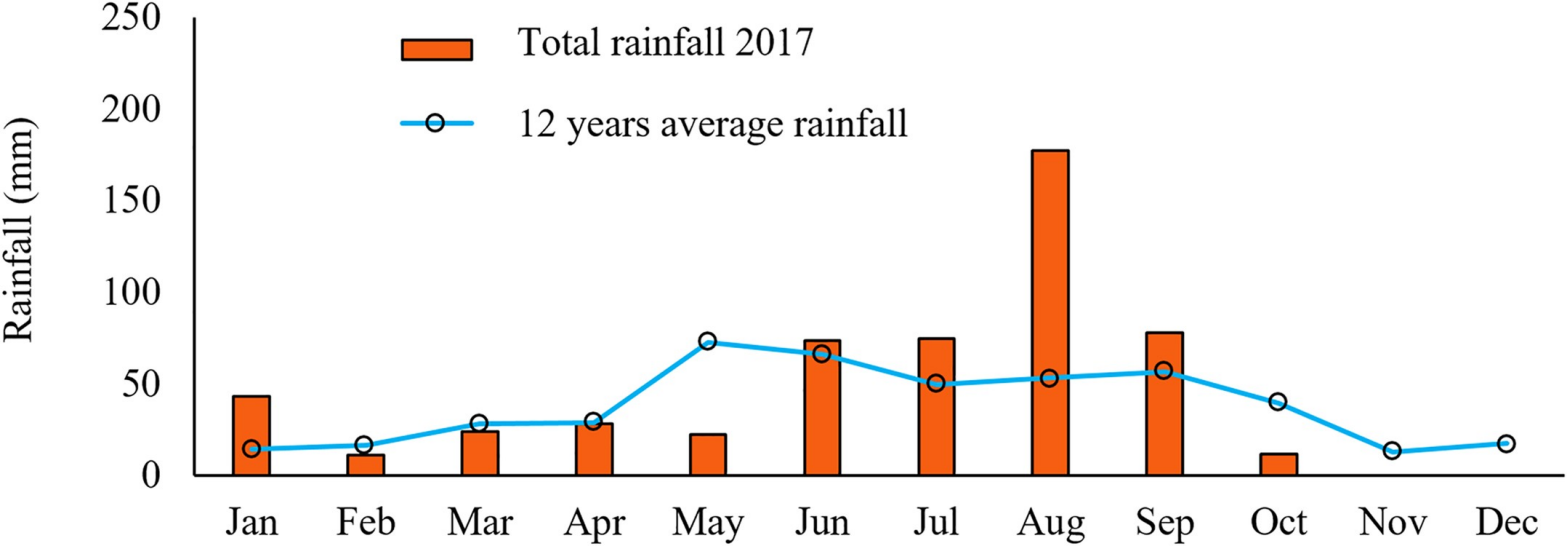
Monthly rainfall for 2017 and the 12-year average for the area at the study site in Hale County, Texas.

The rainfall data for the fields were collected from the West Texas Mesonet located 8 km south of the fields. Total rainfall of 506 mm was received in the area for 2017, with the largest rainfall event of 177 mm recorded in August (Fig 3).

#### Soil sample collection and analysis

**Fig 4:**
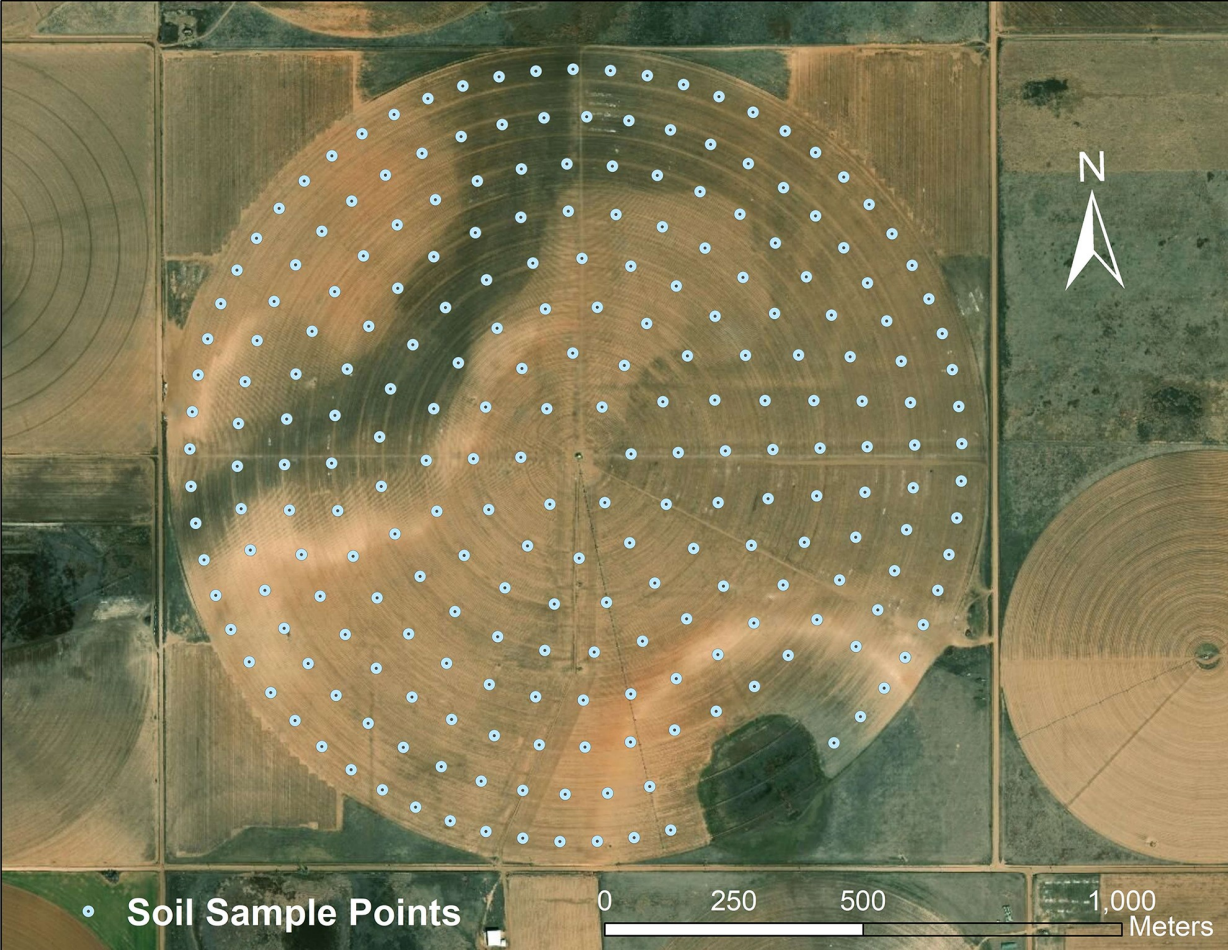
Soil sampling design for a 194-ha farm in Hale County, Texas in 2017 (basemap source: Esri, DigitalGlobe, GeoEye, i-cubed, USDA FSA, USGS, AEX, Getmapping, Aerogrid, IGN, IGP, swisstopo, and the GIS User Community).

Soil samples were collected in April 2017 in a grid using a soil sampling unit (Fig 4). A total of 212 samples were collected from the farm at the density of one sample per 0.8 ha. Soil sampling in and around the playa lake in the southeast part of the farm was not considered for data collection. Composite soil samples, each with three cores, were collected at 0‒15 cm and 15‒30 cm depths. Laboratory analysis was carried out to determine soil particle size using a modified hydrometer method [21].

#### Apparent soil electrical conductivity

Apparent soil electrical conductivity (EC_a_) data were collected using an EC Mapping System (Model 3100, Veris Technologies, Salina, Kansas) equipped with a differential global positioning system (DGPS) receiver. This system consists of a Wenner array and records EC_a_ in mS m^-1^ by electrical resistivity at a shallow depth (0–30 cm) and a deep depth (0–90 cm) simultaneously. The shallow and deep readings are abbreviated as EC_a_sh_ and EC_a_dp_, respectively. Data were collected every 3–5 m along transects spaced ~15 m apart, resulting in 200‒300 readings per hectare. The point data were interpolated to 4-m grids using the Kriging procedure in the Geospatial Analyst of ArcGIS (ESRI, Redlands, CA).

#### Elevation and slope

Elevation data were collected using a real-time kinematic (RTK) GPS receiver with an accuracy of 1 cm, using a planter during planting on transects spaced ~15 m, resulting in one data point every 15 m. The digital elevation point data were used to generate a digital elevation model (DEM) for the field surface. The DEM was developed by interpolating the point data to 4 m raster grids using the Kriging procedure in Geospatial Analyst of ArcGIS. The slope for the field was derived from this DEM.

#### Yield monitor data

Seed cotton yield was collected using strippers equipped with optical yield monitors (Model AG700, AGRIplan, Stow, MA), and a DGPS (Differential GPS) with sub-meter accuracy. The swath of the harvester was 8 m (eight rows), and logging intervals were one or two seconds, resulting in a yield data point every 2 or 4 m. Yield data points collected were subjected to post-harvest screening to remove errors. Yield readings at very low speed (1.6 km h^-1^) were removed as they corresponded to the frequent stops of the harvesting stripper during unloading of cotton, removal of weeds in the harvester header, and other reasons. Similarly, readings associated with high speed (>9.7 km h^-1^) were also removed because they were corresponding to conditions when the stripper operated without harvesting cotton. The yield data points were then interpolated into a 4-m raster grid using the Geostatistical Analyst routine (Kriging) in ArcGIS.

#### Data aggregation and analysis

A polygon shapefile containing the subplots was created using ArcGIS. These polygons were superimposed on the 4-m grids of yield, elevation, slope, sand content, clay content, and EC_a_. The Zonal Statistics routine in the Spatial Analyst extension in ArcGIS was used to calculate and extract the arithmetic average of each data layer for each polygon. Summary statistics for cotton lint yield, clay content, sand content, EC_a_, elevation, and slope were determined using the R program [22]. The normality for each data set was tested using the Shapiro-Wilk test. Simple correlations between yield and other variables were obtained using the *cor* function. The relationship between cotton lint yield and sand content, clay content, EC_a_, elevation, and slope were analyzed with linear mixed effect models [23] using the function from the package NLME [24]. The model included cotton lint yield as the response variable, irrigation (n = 3), soil and topographical properties as fixed effects, and block (n = 2) as a random effect. The variable selection for the yield response model in each field was made using the backward stepwise regression. Autocorrelation can obscure statistical testing because it violates the assumption of independently and identically distributed errors of most standard statistical procedures. Hence, the regression model was adjusted for spatial autocorrelation by incorporating a spherical semivariogram model with the restricted maximum likelihood (REML) method [23].

## Results and discussion

### Variability in soil properties

**Fig 5:**
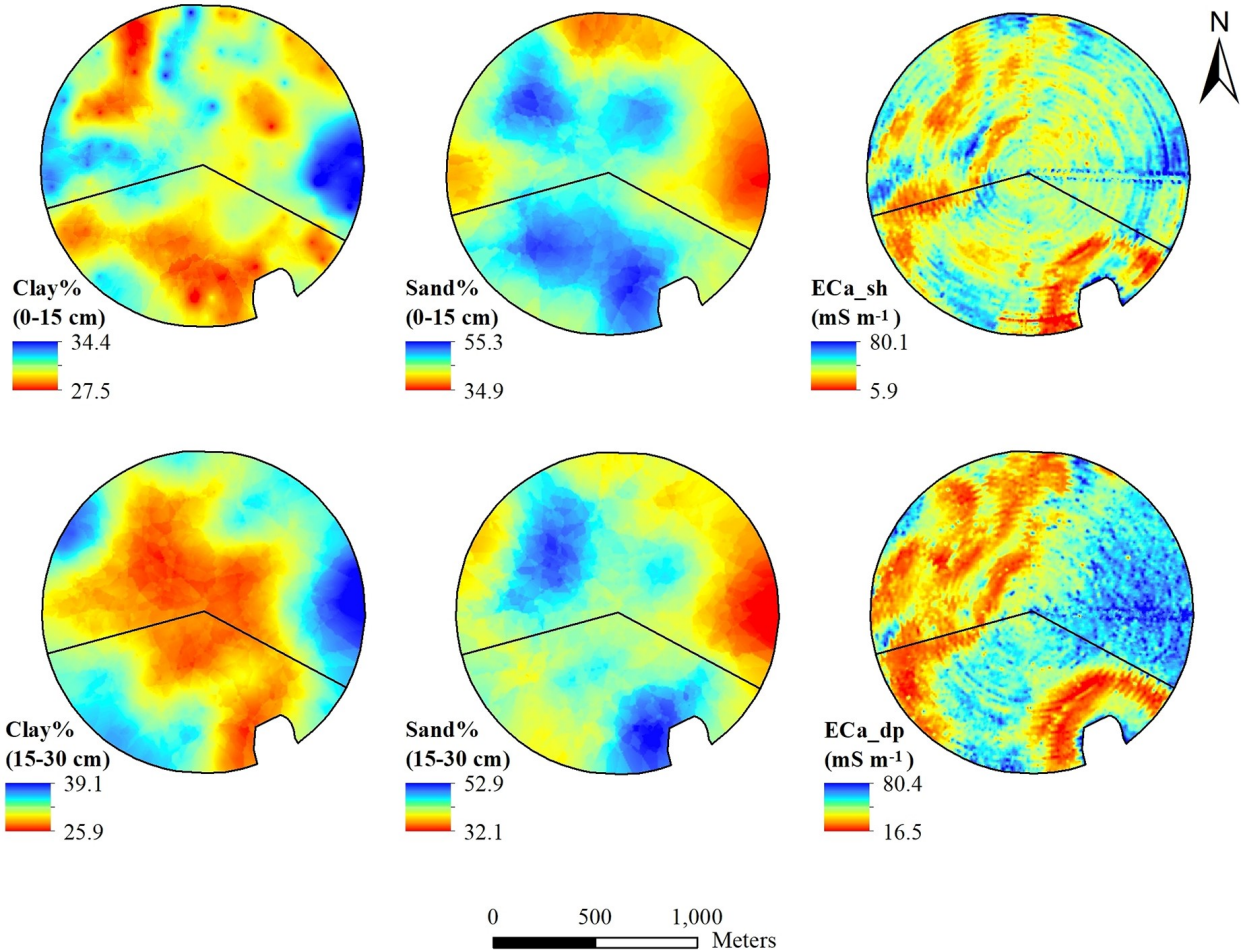
Soil texture and EC_a_ variability for two fields within a 194-ha farm in Hale County, Texas.

Fig 5 shows the spatial variability of soil properties. The mean clay content was 29% and 31% in the North Field for 0‒15 cm and 15‒30 cm, respectively (S1 Appendix). In the South Field, mean clay content was 27% and 31% for 0‒15 cm and 15‒30 cm, respectively. The range and standard deviation for clay content were higher in the subsurface layer for both fields. The mean sand content was higher in the South Field for both layers compared to the North Field. In general, sand content in the surface layer was highly variable across the field with a range of 22.3%. The mean shallow (0‒30 cm) and deep (0‒90 cm) EC_a_ values were 46 mS m^-1^ and 32.8 mS m^-1^, respectively, in the North Field. These values are higher compared to the South Field. Both the standard deviation and range were higher for deep EC_a_ in the North Field compared to the South Field.

#### Variability in topography

**Fig 6:**
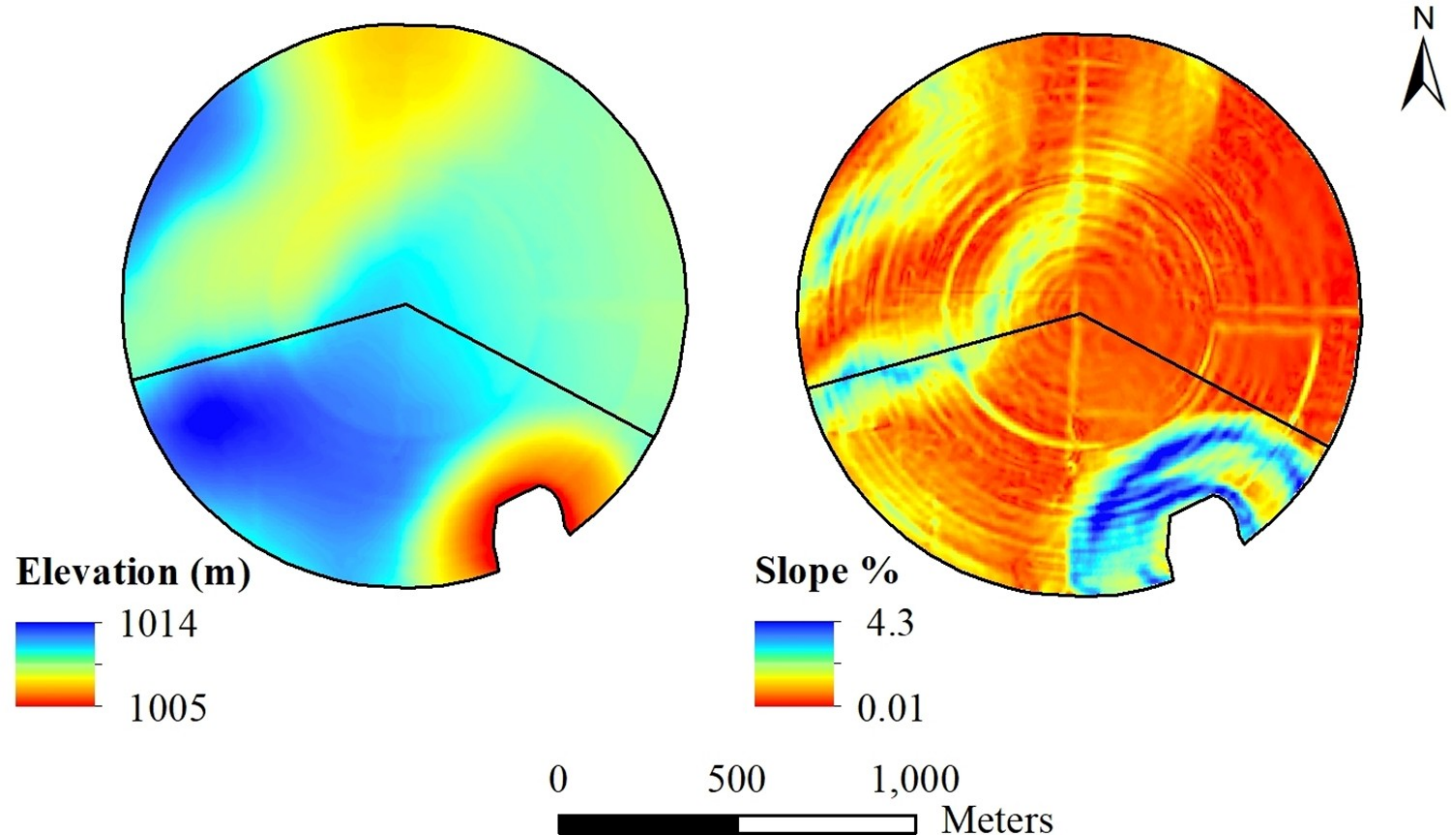
Variation in elevation and slope for two fields within a 194-ha farm in Hale County, Texas.

Elevation across the entire field ranged from 1005 m to 1014 m, and slope ranged from 0% to 4.3% (Fig 6). Elevation in the North Field ranged from 1008 m to 1012 m, and for the South Field the range was 1005 m to 1014 m (S1 Appendix). The South Field had a higher variation in slope (0.1% to 3.2%) as compared to the North Field (0.1% to 2.0%) due to the presence of the playa lake.

#### Cotton lint yield variability

**Fig 7:**
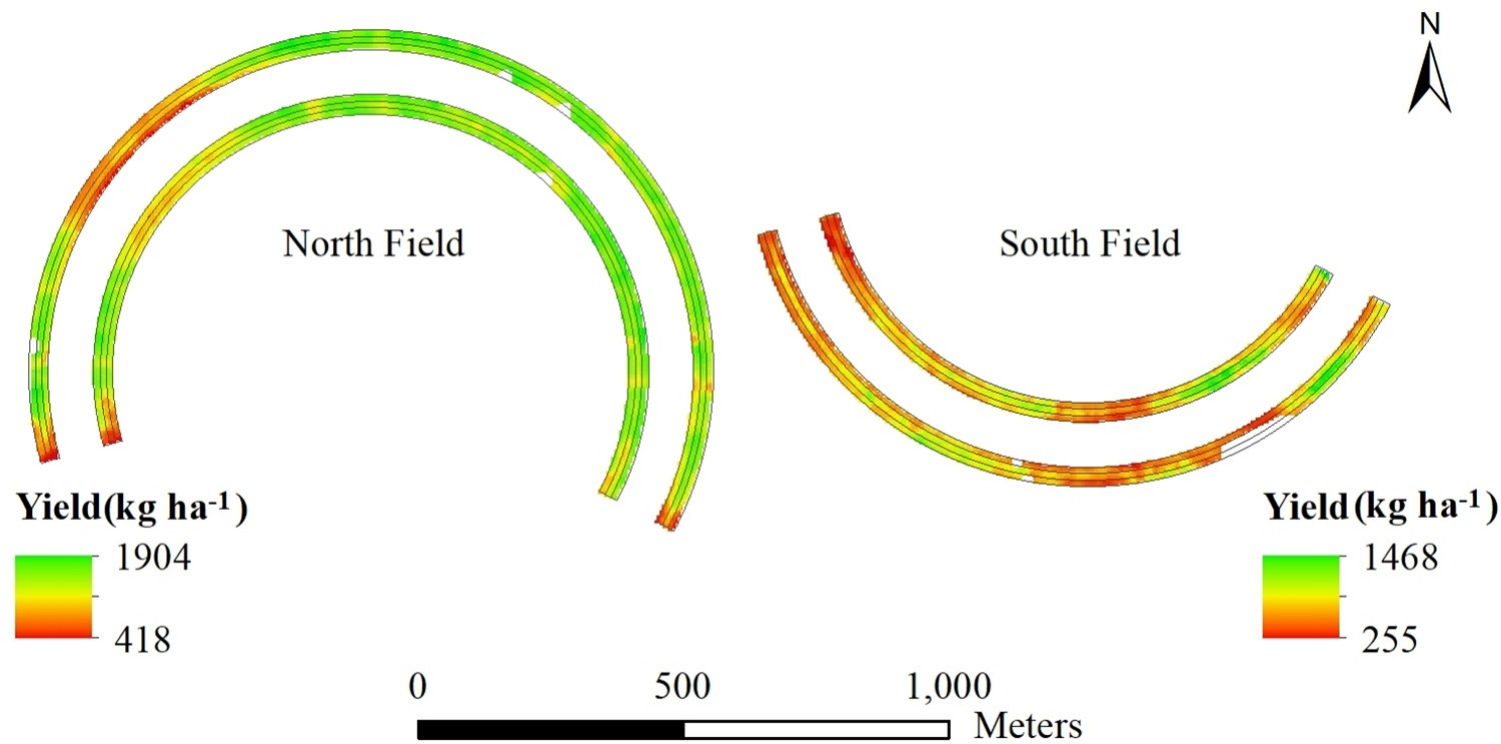
Variation in cotton lint yield for two fields within a 194-ha farm in Hale County, Texas, in 2017.

Cotton yield showed spatial variability across the experimental strips in both fields (Fig 7). Cotton lint yield ranged from 500 kg ha^-1^ to 1892 kg ha^-1^ in the experimental areas in the North Field and from 179 kg ha^-1^ to 1170.5 kg ha^-1^ in the South Field (S1 Appendix). Mean yield was higher for the North Field (1451 kg ha^-1^) compared to the South Field (664 kg ha^-1^). The yield was highly variable in the North Field as indicated by a high standard deviation in the North Field (297.3 kg ha^-1^) as compared to the South Field (186.6 kg ha^-1^). However, the median yield of 1463.1 kg ha^-1^ in the North Field indicates a higher yield in most of the experimental areas of the North Field as compared to the South Field that had the median yield of 613.5 kg ha^-1^. In the late season of 2017, low temperature and high rainfall resulted in low yield for Deltapine variety, which might be one of the reasons for the significant yield difference between these two fields. This situation was widespread for Lubbock and Hale Counties and was reported by all cotton growers and researchers in the area [25].

#### Relationship between sand content, clay content, EC_a_ and topography

Table 1 shows correlations among clay content, sand content, EC_a_, elevation, and slope. EC_a_ was positively correlated with clay content and negatively related to sand content. EC_a_ was reported to be strongly related to topsoil clay and sand content [26] and also affected by water content [27]. The bulk density of soil and the presence of Ca^2+^, Mg^2+^, Na^+^, CEC, silt, and soluble salts also affect EC_a_ [28, 29]. In the North Field, clay content was higher, indicating the potential of soil to hold more water. Since EC_a_ is a function of soil texture and water content, this might be the reason for the higher correlation of EC_a_ with clay content in the North Field as compared to the South Field. Similar phenomena were reported in a study conducted by Bronson et al. (2005) in the SHP region [28]. Elevation and slope had a significant correlation with soil texture and EC_a_ in these two fields. The correlation of elevation to soil properties was higher in the North Field as compared to the South Field. This might be due to higher variation of elevation in the South Field. The slope was positively correlated with sand content and negatively correlated with clay content in both fields. This indicates that areas with high slopes tend to have higher sand content and lower clay content. This might be due to the runoff of water together with smaller soil particles such as clay from high slope areas to the playa lake or other depressed areas on the farm. Further, this could also be the reason for a negative correlation between slope and EC_a_ in both fields since EC_a_ is a function of soil texture and water content. Similar results were found in another study conducted in this area [30].

**Table 1:** Correlations between sand content, clay content, EC_a_, elevation and slope for two fields within a 194-ha farm in Hale County, Texas, in 2017.

Variable	Sand_L1	Sand_L2	Clay_L1	Clay_L2	EC_a_sh_	EC_a_dp_	Slope
------------------------North Field (n = 246) ------------------------							
Sand_L2	0.69***						
Clay_L1	-0.83***	-0.58***					
Clay_L2	-0.51**	-0.90***	0.48***				
EC_a_sh_	-0.63***	-0.64***	0.59***	0.64***			
EC_a_dp_	-0.52***	-0.76***	0.40***	0.73***	0.73***		
Slope	0.63***	0.47***	-0.50***	-0.32***	-0.53***	-0.59***	
Elevation	0.21***	-0.24***	-0.16**	0.34***	0.20***	0.38***	0.27***
------------------------South Field (n = 141) -------------------------							
Sand_L2	0.40***						
Clay_L1	-0.44***	-0.46***					
Clay_L2	-0.24**	-0.82***	0.24**				
EC_a_sh_	-0.34***	-0.44***	0.30***	0.55***			
EC_a_dp_	0.01	-0.62***	0.22**	0.56***	0.70***		
Slope	0.12	0.80***	-0.21**	-0.66***	-0.50***	-0.81***	
Elevation	-0.03	-0.82***	0.24**	0.83***	0.28***	0.51***	-0.70***

***, **, *: significant at 0.001, 0.01, 0.05 probability levels, respectively; Sand_L1: Sand content (%) for 0–15 cm; Sand_L2: Sand content (%) for 15–30 cm; Clay_L1: Clay content (%) for 0–15 cm; Clay_L2: Clay content (%) for 15–30 cm; EC_a_sh_: EC_a_ (mS m^-1^) for 0–30 cm; EC_a_dp_: EC_a_ (mS m^-1^) for 0–90 cm.

#### Cotton lint yield in relation to irrigate rates, soil physical properties and topography

The semivariogram analysis for cotton lint yield indicated the presence of autocorrelation among the plots. The best-fitted semivariogram for the yield in the North Field was a spherical model with a range of 489.95 m. The nugget to sill ratio of 0.13 indicated the presence of strong spatial dependence. The best-fitted semivariogram for the yield in the South Field was a Gaussian model with a range of 173 m. The nugget to sill ratio of 0.31 indicated the presence of strong spatial dependence. Hence, the multiple regression model was updated to account for spatial autocorrelation. The yield was autocorrelated at a smaller range in the South Field than the North Field, possibly due to higher variability of soil and topographical properties in the South Field. The AIC (Akaike’s Information Criteria) and BIC (Bayesian Information Criterion) were smaller for the updated model. The log-likelihood for the updated model was higher compared to the model that did not account for autocorrelation. AIC, BIC, and log-likelihood are model assessment criteria. The model with smaller AIC and BIC but higher log-likelihood values are considered as the preferred model [31, 32]. This indicates that the model updated for spatial analysis was a better fit compared to the non-updated model and hence had a greater chance to observe the data. Model outputs for the North and the South Field are shown in Table 2. For the North Field, the three-way interaction between irrigation, slope, and clay content had a significant effect on the yield. For the South Field, the interaction of irrigation with sand content and slope had a significant effect on the yield.

**Table 2:** Linear mixed-effects model for predicting cotton lint yield using irrigation rates, soil properties, and topography in two fields within a 194-ha farm in Hale County, Texas in 2017.

Variable	Coefficient	Std. Error	p-value
------------------------North Field-------------------------------			
Irrigation	-2.39	1.83	0.19
Clay_L1	-87.83	60.45	0.15
Sand_L1	-21.84	11.85	0.06
Sand_L2	-29.49	12.18	0.02
Elevation	-208.57	72.73	<0.01
Slope	-4540.47	2112.29	0.03
Irrigation: Clay_L1	0.10	0.06	0.10
Irrigation: Slope	7.31	2.66	<0.01
Clay_L1:Slope	148.39	75.61	0.05
Irrigation:Clay_L1:Slope	-0.25	0.09	<0.01
------------------------South Field------------------------------			
Irrigation	17.64	3.2	<0.01
Clay_L2	-0.36	22.67	0.98
Sand_L1	266.44	49.275	<0.01
Sand_L2	-12.74	13.25	0.33
Elevation	-66.56	28.72	0.02
Slope	3662.50	1478.40	0.01
Irrigation: Sand_L1	-0.35	0.064	<0.01
Irrigation: Slope	-5.34	1.96	<0.01
Sand_L1:Slope	-72.17	28.89	0.01
Irrigation:Sand_L1:Slope	0.10	0.04	<0.01

Sand_L1: Sand content (%) for 0–15 cm; Sand_L2: Sand content (%) for 15–30 cm; Clay_L1: Clay content (%) for 0–15 cm; Clay_L2: Clay content (%) for 15–30 cm; EC_a_sh_: EC_a_ (mS m^-1^) for 0–30 cm; EC_a_dp_: EC_a_ (mS m^-1^) for 0–90 cm.

**Fig 8:**
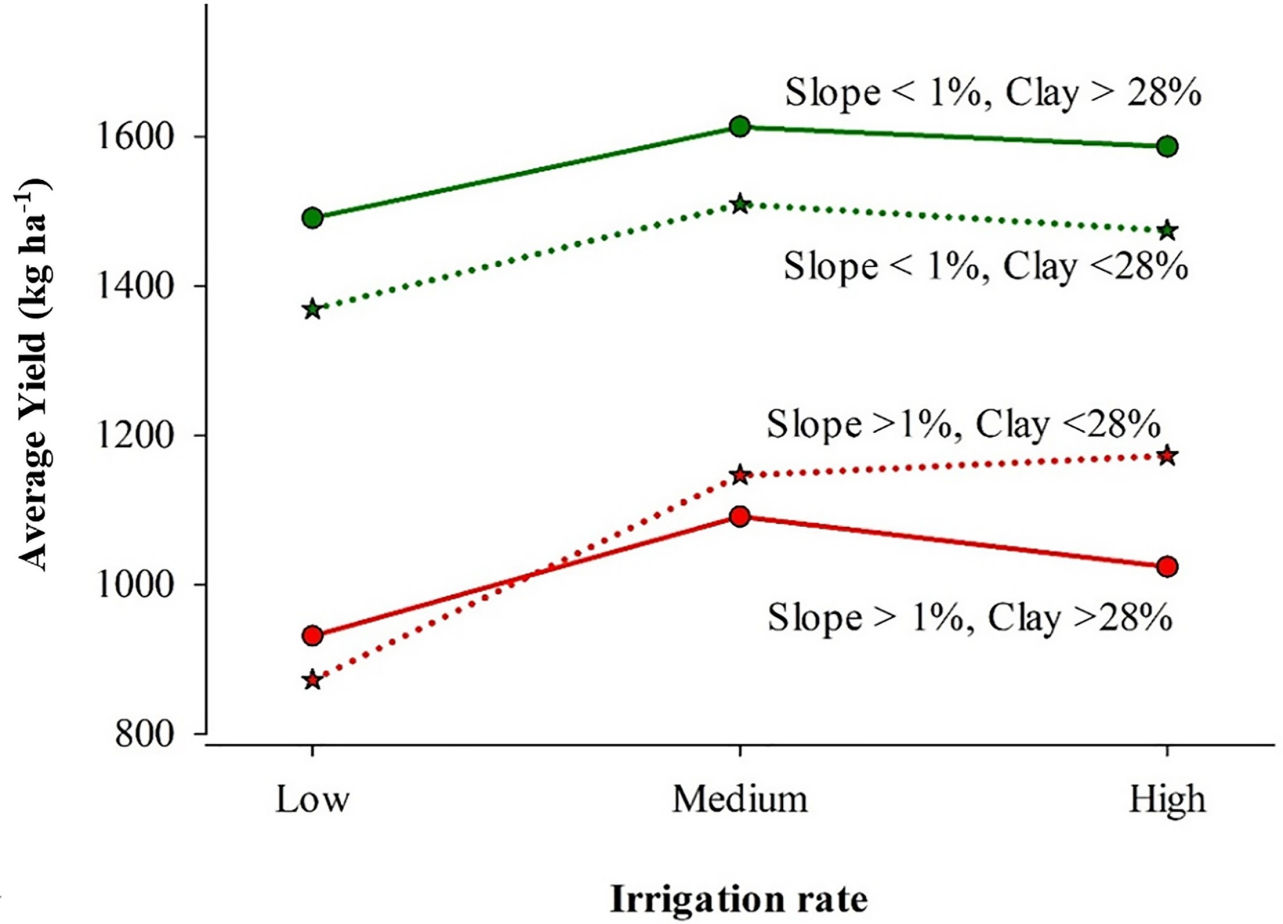
Interaction effects between irrigation rate, clay content, and slope on cotton lint yield in a 122-ha field in Hale County, Texas.

The interaction between irrigation and clay content and slope had a significant effect on yield variation for the North Field (Fig 8). For areas with a low slope (<1%), the yield was higher for areas with higher clay content, and the response pattern of yield to different irrigation rates was similar. However, applying high irrigation instead of medium did not appear to contribute towards incremental yield. For areas with a higher slope (>1%), clay content in the soil had a significant influence on the response of yield to irrigation rates. Evidently, in areas with lower clay content and higher slope, applying a higher irrigation rate improved yield slightly as compared to the medium irrigation rate. Since these areas have low water holding capacity, irrigation water might have helped to boost crop growth and final yield. Contrarily, in areas with higher slope and clay content, applying high irrigation is detrimental since the yield has been found to decrease as compared to when medium irrigation was applied. This result was similar to another study that found that during wetter years, the yield was negatively correlated with clay content [33]. This indicates that for a field with a slope of less than 1% and with clay content higher than 28%, irrigating at a medium rate rather than a higher rate can produce optimum yield for years with high rainfall. Similarly, in high slope areas (>1%), the yield was lower and high irrigation produced higher yield, indicating that applying high irrigation was only better for areas with lower clay content. Hence, water use can be optimized in these areas. However, the situation might be different for years with lower rainfall.

**Fig 9:**
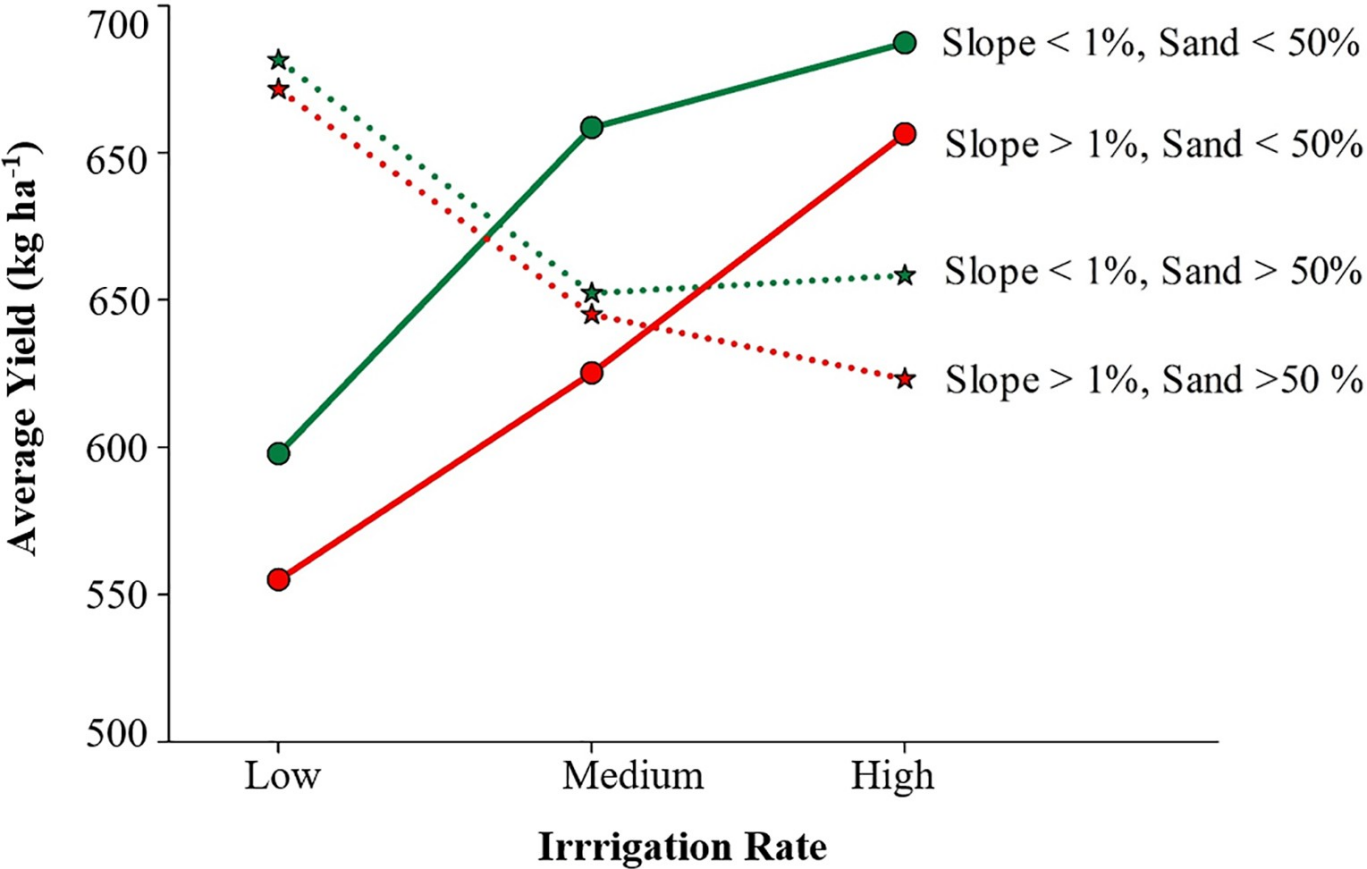
Interaction effects between irrigation rates, sand content (0–15 cm), and slope on cotton yield in a 72-ha field in Hale County, Texas in 2017.

The interaction between sand content, irrigation, and slope had a significant effect on yield for the South Field (Fig 9). The slope ranged from 0% to 4%, and the sand content ranged from 45% to 60%. This resulted in variable yield at different sand contents and slope positions for different irrigation rates. For areas with a low slope (<1%) and comparatively lower sand content (<50%), the yield was greater for high irrigation rates and less for low irrigation. This suggests that for these areas, the application of more water could lead to increased yield. However, for the areas with sand content greater than 50% and slope greater than 1%, the yield was low, and low irrigation resulted in greater yield than at high irrigation. Another study also showed greater lint yields in bottom slopes than side slopes, due to greater runoff of nutrients together with excess water from the soil surface [34]. Another research on nitrogen response in cotton as affected by irrigation level showed that, with high irrigation and rainfall, there is a possibility that nutrients could percolate with water and result in decreased yield [35]. This might be one of the causes of low yields in low slope areas in the South Field with high sand content. Therefore, low irrigation might have prevented the leaching of nutrients and yield more than other irrigation rates. This indicates that there is a potential to improve water and nutrient use efficiencies by applying lower irrigation rates in these areas. Another study conducted in a spatially variable field in the SHP also showed that a very sandy soil with high slope had low yield when given the same amount of irrigation water, fertilizer, seed population, and the same management as other soils [36]. In general, for the South Field, high irrigation in areas with low slope and sand might increase yield, and applying low irrigation in areas with high slope and sand can save water. This may not apply to all years with different weather conditions. However, farmers can prescribe the amount of irrigation with a similar season like in this study to obtain potential water savings.

**Fig 10:**
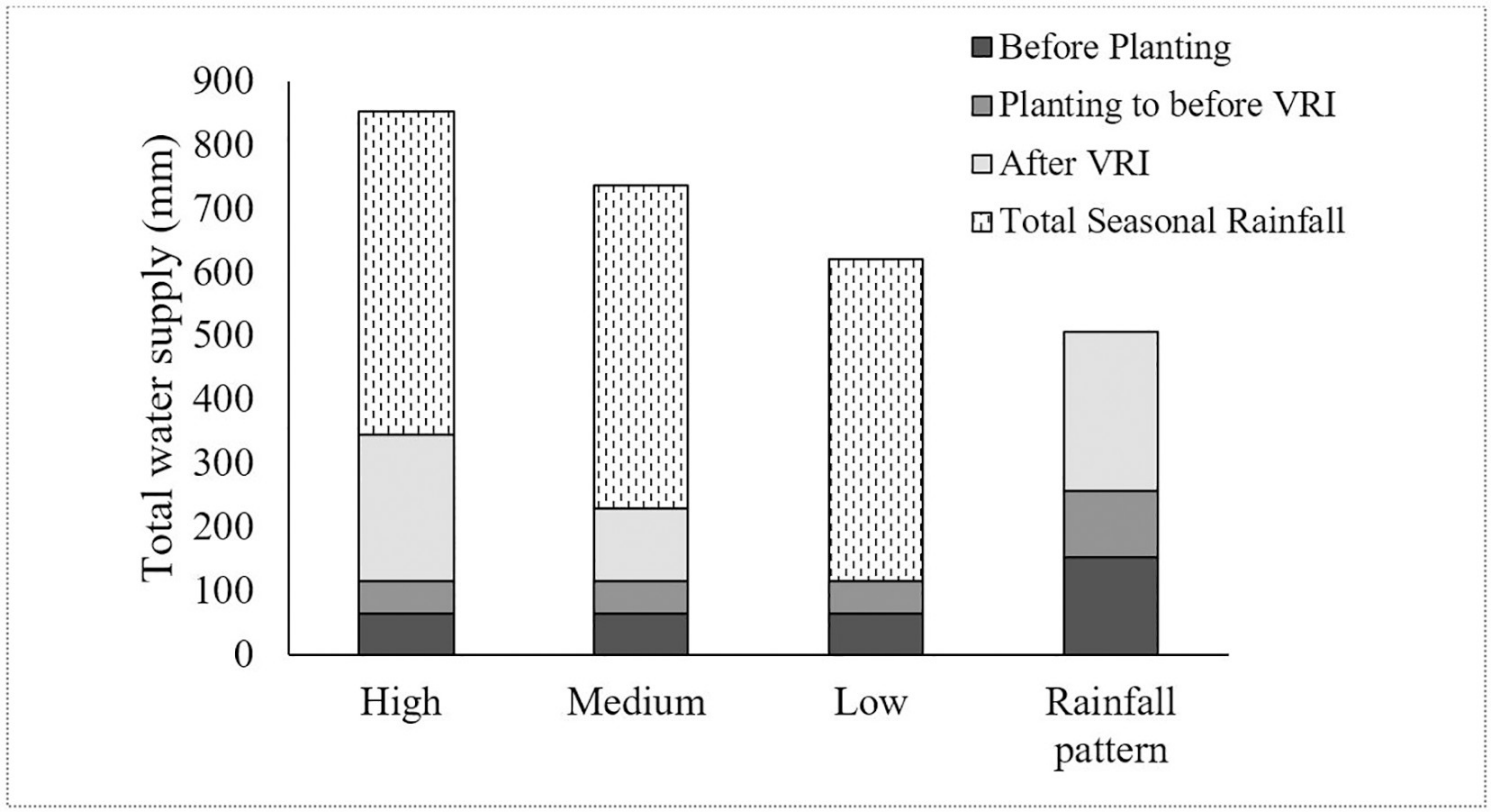
Pre- and in-season irrigation and rainfall for a 194-ha farm in Hale County, Texas in 2017.

For the North Field, the effect of irrigation rates on yield was not significant. This could be due to greater than average rainfall distributed throughout the growing season. The average potential crop evapotranspiration in this area is about 846 mm [1]. In a 12-year study conducted in this area, an irrigation input of 580 mm or total water application of 740 mm was needed to produce lint yield ranging from 855 kg ha^-1^ to 1630 kg ha^-1^ [37]. In this study, the total seasonal rainfall was 506 mm. Total water applied, including rainfall, was 852 mm for high, 736 mm for medium, and 621 mm for low irrigation rate treatments (Fig 10). Hence, the high seasonal rainfall amount masked the effect of irrigation rate on cotton yield, resulting in no significant difference in yield among the three irrigation rates. However, in the South Field, the irrigation rate had a significant effect on the yield, possibly due to the high variation in the soil and topographic properties in the South Field.

**Fig 11:**
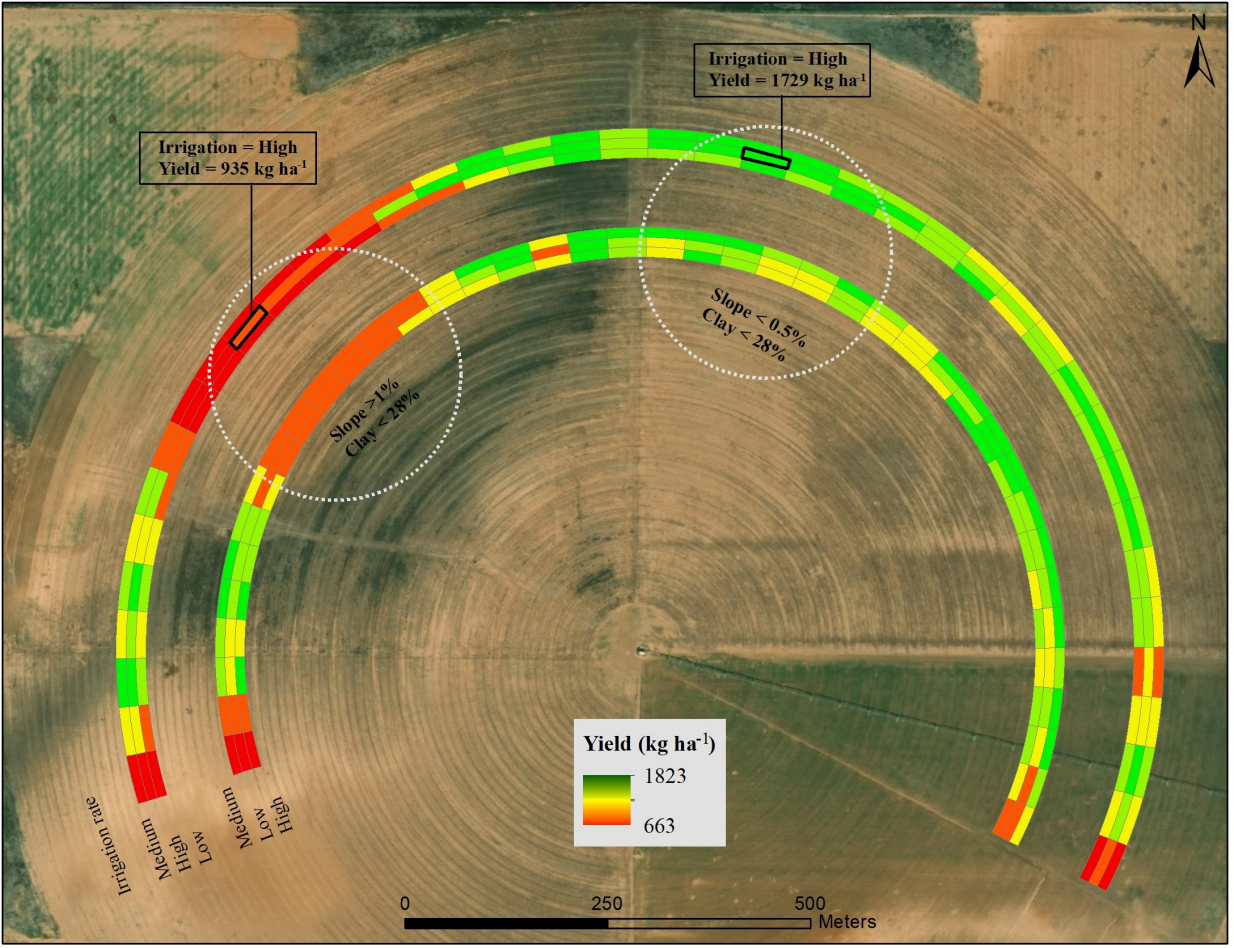
Cotton lint yield variability for three irrigation rates in a 122-ha field within a 194-ha farm at two areas with variable clay content (%) and slope (%) in Hale County, Texas in 2017. (Basemap source: Esri, DigitalGlobe, GeoEye, i-cubed, USDA FSA, USGS, AEX, Getmapping, Aerogrid, IGN, IGP, swisstopo, and the GIS User Community).

Fig 11 shows an example of the effect of soil property and slope on yield for the North Field. The yield was higher at area-A with clay content less than 28% and slope less than 0.5%, compared to area-B where clay content was less than 28%, but the slope was greater than 1%. For the high irrigation rate, the yield at area-A was 1729 kg ha^-1^, compared to the yield of 935 kg ha^-1^ for area-B. This shows that for the same irrigation rate, yield varied at different landscape positions.

#### Challenges with multiple-year experiment

We attempted to repeat this study after 2017. However, due to some farm management issues and weather-related hazards, the experiment and data collection could not be implemented from 2018 to 2020. In addition, the well capacity dropped from 800 gallons/minute in 2017 to only 400 gallons/minute in 2020, making it impossible to replicate this study in multiple seasons under the same experimental settings. However, this single-year study serves as an important basis for precision irrigation in this region. The producer has adopted a site-specific irrigation strategy by only irrigating the northeast section of this farm based on this study and historical yield data. This also demonstrates the immanent need for site-specific irrigation management under depleting water resources in this and other similar regions. Nevertheless, similar studies in other fields are required to evaluate and validate cotton yield as affected by soil and topographic factors before implementing site-specific water management.

## Conclusion

Cotton lint yield variability in relation to three irrigation rates, soil physical properties, and topography was evaluated in two fields within a 194-ha commercially managed farm in the Southern High Plains in 2017. A statistical model was developed to predict yield response to irrigation rates, soil texture, EC_a_, topography, and their interactions. This model showed that the effect of irrigation rate on cotton lint yield depended on its interaction with soil physical properties and topography. Irrigation rates had no significant effect on cotton lint yield, possibly due to greater than long-term average in-season rainfall in 2017. However, cotton lint yield response to a given irrigation rate varied with different landscape positions and soil physical properties. In general, the yield was greater for areas with high clay content, low sand content, and low slope, and high irrigation rates in these areas produced a higher yield. Conversely, in areas with high sand content and high slope, yield response to irrigation rates was not significant. This yield response pattern suggests there is a potential for site-specific water management. For example, applying less irrigation in areas with high sand content and high slope could save water without reducing yield. Hence, the model developed in this study can help determine management zones in the field based on yield response to irrigation rates that could be utilized for variable rate irrigation.

This study was conducted in full-scale commercial fields that represent the common water management practice in this region. The results of this study can identify and address issues at the operational scale of VRI, potentially leading to an increased involvement of farmers and their interest in adoption. However, more studies in multiple years with different weather conditions are required to determine the right irrigation rates at different landscape positions based on the model developed in this study. Further studies on economic analysis are required to assess the viability of VRI for producers to adopt this technology. The incorporation of other yield influencing factors, such as soil organic matter, can improve the model for optimized water management.

## Supporting information

S1 Appendix S1 Table. Summary statistics of cotton lint yield, soil physical properties, and topographic attributes for two fields in Hale County, Texas, in 2017.
(DOCX)

S1 Data (XLSX)

S1 Metadata (TXT)

S1 Raw data (DOC)

S1 File (ZIP)

S2 File (ZIP)

S3 File (ZIP)

S4 File (ZIP)

S5 File (ZIP)

S6 File (ZIP)
