# Supplementary material for: Effects of irrigation rates on cotton yield as affected by soil physical properties and topography in the southern high plains
Source: PLoS One. 2021 Oct 26;16(10):e0258496. doi: 10.1371/journal.pone.0258496 (PMC8547627; doi:10.1371/journal.pone.0258496)
Supplement: S1 Appendix — Summary statistics of cotton lint yield, soil physical properties, and topographic attributes for two fields in Hale County, Texas, in 2017. (DOCX) [file pone.0258496.s001.docx]

# Appendix

**S1 Table. Summary statistics of cotton lint yield, soil physical properties, and topographic attributes for two fields in Hale County, Texas, in 2017.**

| Variable | Min | Max | Range | Median | Mean | SD | CV |
| --- | --- | --- | --- | --- | --- | --- | --- |
| -------------------------------------------------------- North Field -------------------------------------- | | | | | | | |
| Yield (kg ha^-1^) | 501 | 1893 | 1392 | 1539 | 1463 | 297 | 0.20 |
| Sand_L1 (%) | 35.2 | 57.5 | 22.3 | 43.0 | 44.4 | 4.9 | 0.11 |
| Sand_L2 (%) | 32.8 | 51.7 | 18.9 | 42.1 | 42.1 | 4.4 | 0.10 |
| Clay_L1 (%) | 25.3 | 32.6 | 7.3 | 28.6 | 28.8 | 1.6 | 0.05 |
| Clay_L2 (%) | 26.4 | 37.7 | 11.3 | 30.7 | 31.1 | 2.7 | 0.08 |
| EC_a_dp_ (mS m^-1^) | 22.9 | 73.5 | 50.7 | 44.1 | 46.4 | 13.1 | 0.28 |
| EC_a_sh_ (mS m^-1^) | 17.7 | 47.0 | 29.3 | 33.6 | 32.8 | 6.2 | 0.19 |
| Elevation (m) | 1008.3 | 1011.8 | 3.5 | 1010.2 | 1010.0 | 0.8 | 0.001 |
| Slope (%) | 0.1 | 2.0 | 1.9 | 0.4 | 0.6 | 0.4 | 0.66 |
| ---------------------------------------------------------- South Field ------------------------------------ | | | | | | | |
| Yield (kg ha^-1^) | 179 | 1171 | 991 | 604 | 614 | 187 | 0.30 |
| Sand_L1 (%) | 42.8 | 55.6 | 12.8 | 50.3 | 49.9 | 3.3 | 0.06 |
| Sand_L2 (%) | 40.5 | 56.6 | 16.1 | 43.3 | 45.4 | 4.4 | 0.09 |
| Clay_L1 (%) | 25.2 | 29.1 | 3.9 | 26.7 | 26.9 | 1.0 | 0.04 |
| Clay_L2 (%) | 25.9 | 34.2 | 8.3 | 31.5 | 31.0 | 2.5 | 0.08 |
| EC_a_dp_ (mS m^-1^) | 23.5 | 61.9 | 38.4 | 42.3 | 41.0 | 10.8 | 0.26 |
| EC_a_sh_ (mS m^-1^) | 17.5 | 48.5 | 31.0 | 29.9 | 30.3 | 7.1 | 0.23 |
| Elevation (m) | 1006.3 | 1013.9 | 7.7 | 1012.4 | 1011.4 | 2.0 | 0.002 |
| Slope (%) | 0.1 | 3.2 | 3.1 | 1.0 | 1.3 | 0.9 | 0.69 |
| Sand_L1: Sand content % for 0-15 cm; Sand_L2: Sand content (%) for 15-30 cm; Clay_L1: Clay content (%) for 0-15 cm; Clay_L2: Clay content % for 15-30 cm; EC_a_sh_: EC_a_ (mS m^-1^) for 0-30 cm; EC_a_dp_: EC_a_ (mS m^-1^) for 0-90 cm | | | | | | | |
