# Supplementary material for: Effects of irrigation rates on cotton yield as affected by soil physical properties and topography in the southern high plains
Source: PLoS One. 2021 Oct 26;16(10):e0258496. doi: 10.1371/journal.pone.0258496 (PMC8547627; doi:10.1371/journal.pone.0258496)
Supplement: S1 Raw data — (DOC) [file pone.0258496.s004.doc]

**README.TXT (updated 7/01/13)**

*New stations 60 and up are one-minute observations (array 1 format). Format is the same as five-minute array one-minute observations, just one-minute averages with 3-second sampling for most parameters. We are slowly adding 20 ft wind data to all one-minute stations. The average 20 ft one-minute wind speed is the last parameter in the array 1 file...after the pyranometer data.*

Each raw monthly file contains comma-delimited data that is separated into two or three arrays. There are two different file formats: REESE and NON-REESE. The REESE file is strictly for the REES mesonet station. This file contains three arrays: Array 1 has Met. Sensor data, Array 2 has Soil data, Array 3 has Solar data only (pre-2006) and wind data (post-2009). *The NON-REESE file format is the same for all other mesonet stations: Array 1 has Met. Sensor data and Array 2 has Soil data*.

We were forced to use different arrays to accommodate the radio communication system.

**I. NON-REESE file format (Order parameters appear in file)**

*Array ID*: 1 (observation every 5 minutes)

*Day*: Julian (January 1^st^ is 1, etc).

*Local Time*: always local standard time; add one hour for daylight savings time

*Station ID*: see “MesonetID text file for list of datalogger ID’s”

*10-meter Wind Speed* (Scalar value in m/sec)=5-minute average of 3-second samples

10-meter anemometer is an R.M. Young 05103-L Wind Monitor or R.M. Young 05103-L Alpine Wind monitor (new stations): typical values vary from 0 to 40 m/sec. The unit will measure up to 65 m/sec.

*10-meter Wind Speed* (Vector value in m/sec)=5-minute average of 3-second samples

*10-meter Wind Direction* in degrees=5-minute average of 3-second samples

*10-meter Wind Direction*-*Standard Deviation*

*10-meter Wind Speed-Standard Deviation*

*10-meter Wind Speed*= Peak three-second wind gust (m/sec)

*1.5-meter Temperature* in degrees C=5-minute average of 3-second samples

1.5-meter Temperature and Relative Humidity Probe (Vaisala HMP45C). Measurement range of the unit for temperature is –40 to +60 C.

New stations use (Vaisala HMP155) with 14-plate radiation shield (new units only, with one-minute averaging)

*9-meter Temperature (Heat Flux)* in degrees C=5-minute average of 3-second samples

9-meter Temperature Probe is a Campbell Scientific 107-L. Measurement range of the unit is –35 to 50 C

*2-meter Temperature (Heat Flux)* in degrees C=5-minute average of 3-second samples

2-meter Temperature Probe is a Campbell Scientific 107-L.

*1.5-meter Relative Humidity*=5-minute average of 3-second samples.

Vaisala HMP45C with 0-100% RH, non-condensing

Vaisala HMP155 with 0-100% RH, non-condensing (new stations only, one-minute)

*Station Pressure in mb*=add **600** to get correct value in mb (hPa). Our radio is only 8-bit. We have to subtract 600 from the correct value to transmit with the radios. 5-minute average of 12-second samples. Remember to add 600.0 for the correct value…..

*Vaisala PTB220 or PTB330 Digital Barometer* (normal values are around 300 mb (add 600=900 mb) on the caprock; values around 330 mb (930 mb) off the caprock)

Rainfall in inches (total in 1 or 5-minute ob period)

*Hydrological Services TB3 Siphon Tipping Bucket gauge* (total is summed over the 5-minute ob period). All the 5-minute observations must be added to get daily total. Units are 0.01 inches per tip.

*Texas Electronics 525S - Siphon Tipping Bucket gauge (8-inch*)…used on newer on-minute stations (total is summed over the one-minute ob period). All the one-minute observations must be added to get daily total. Units are 0.01 inches per tip.

Dewpoint=5-minute average of 3-second samples

Dewpoint is calculated internally by using the measured parameters.

2-meter Wind Speed=5-minute average of 3-second samples

*R.M. Young Wind Sentry.* Measurement range of 0 to 40 m/sec.

Solar Radiation=CM-3 or SP-Lite Pyranometer…SP-Lite2 Pyranometers on new stations.

*Kipp and Zonen Pyranometers*: Some stations have CM-3 units, most have SP-Lite Pyranometers. All measure accumulated solar radiation in W/m^2. For the daily value, add all the 5-minute observations. All values start at zero for the pyranometer data.

*Kipp and Zonen SP-Lite2 Pyranometer*: New stations after 1/1/2008 are using the new SP-Lite2 units (same output)…slowly replacing SP-Lite units. New stations use one-minute solar radiation value (average of all the 3-second samples in a one-minute period).

20 ft Wind Speed=one-minute average of 3-second samples of wind speed.

R.M. Young Wind Sentry. Measurement range of 0 to 40 m/sec.

(newer stations now have 20 ft wind speed. Will be slowly retrofitting older

stations).

________________________________________________________________________

Array ID: 2 (observation every 15 minutes)

Julian Day

Local Standard Time

Station ID: (see MesonetID file for datalogger ID’s)

Natural Soil Temperature-5cm depth: degrees C

*Campbell Scientific 107-L Temperature Probe* (Measurements range from –35 to +50 C). All soil temperature probes are the same.

Natural Soil Temperature-10cm depth: degrees C

Natural Soil Temperature-20cm depth: degrees C

Bare Soil Temperature-5cm depth: degrees C

Bare Soil Temperature-20cm depth: degrees C

Water Content Reflectometer-5 cm depth (consider units a %)

*Campbell Scientific Water Content Reflectometer 615-L or 616-L (new stations)* . Consider units as a % (with 0.5 being saturated). Equations for each station are based on soil type.

Water Content Reflectometer-20 cm depth (consider units a %)

Water Content Reflectometer-60 cm depth (consider units a %)

Water Content Reflectometer-75 cm depth (consider units a %)

Leaf Wetness Sensor (%)

*Campbell Scientific Leaf Wetness Sensor 237-L*. Units are a %, 0-100%. Installation height is 24 inches above ground level. Simulates moisture droplets on mature cotton plants.

Battery Voltage (volt reading; may vary from about 9 to 14V)

Program Signature (random number assigned during program changes)

**II. REESE file format (Order parameters appear in the file; All descriptions are the same as above except where noted).**

Array ID: 1 (observations taken every 5 minutes)

Julian Day

Local Standard Time

Station ID

10-meter Wind Speed (Scalar value in m/sec)=5-minute average of 3-sec values

10-meter Wind Speed (Vector value in m/sec)=5-minute average of 3-sec values

10-meter Wind Direction in degrees=5-minute average of 3-sec values

10-meter Wind Direction-STD Deviation

10-meter Wind Speed-STD Deviation

10-meter Wind Speed-Peak 3-second wind gust (m/sec)

1.5-meter Temperature in degrees C

9-meter Temperature (Heat Flux) in degrees C

2-meter Temperature (Heat Flux) in degrees C

1.5-meter Relative Humidity

Station Pressure in mb: Add 600 to get correct value

Rainfall in inches (total of 5-minute ob period)

Dewpoint in degrees C=5-minute average of 3-sec values

2-meter Wind Speed in m/sec=5-minute average of 3-sec values

Array ID: 2 (observations every 15 minutes)

Julian Day

Local Standard Time

Station ID

Natural Soil Temperature-5 cm in deg. C

Natural Soil Temperature-10 cm in deg. C

Natural Soil Temperature-20 cm in deg. C

Bare Soil Temperature-5cm in deg. C

Bare Soil Temperature-20cm in deg. C

Water Content Reflectometer-5cm depth (consider units as %)

Water Content Reflectometer-20cm depth (consider units as %)

Water Content Reflectometer-60cm depth (consider units as %)

Water Content Reflectometer-75cm depth (consider units as %)

Leaf Wetness Sensor (%)

Battery Voltage (V)

Program Signature

Array ID: 3 (observations every 5 minutes) – ***through 8/1/2005….only 2 arrays from 8/1/2005 to 3/9/2009. Three arrays begin again on 3/9/2009 for array 3 wind data only.***

Julian Day

Local Standard Time

Station ID

NRLite radiometer (W/m^2)

*Kipp and Zonen NRLite Radiometer*: measures incoming and outgoing radiation. Values will be negative at night and positive during the day unless cloudy (units in W/m^2).

SPLite Pyranometer (W/m^2)

*Kipp and Zonen SP-Lite Pyranometer*: measures incoming radiation (accumulated W/m^2 from 0 to whatever)

Licor Pyranometer (W/m^2)

*Licor Silicon Pyranometer*: Same measurement values as SP-Lite Pyranometer (0 to whatever accumulated W/m^2)

CM21 Pyranometer (W/m^2)

*Kipp and Zonen CM21 Pyranometer*: Main pyranometer for mesonet calibration: measures accumulated W/m^2; 0 to whatever)

CM3 Pyranometer (W/m^2)

*Kipp and Zonen CM3 Pyranometer*: Same measurement values as SP-Lite Pyranometer

Experimental rain gauge in inches (not always present in data)

Currently, a Texas Electronics tipping bucket gauge. Varies from time to time as we test out different rain gauges for vendors. Values are 0.01 increments with total accumulation in 5-minute periods.

Array 3 for WIND data only for REES station (beginning 3/9/2009):

Julian Day

Local Standard Time

Station ID

*10-meter Wind Speed* (Scalar value in m/sec)=5-minute average of 3-second samples

10-meter anemometer is an R.M. Young 05103-L Wind Monitor

typical values vary from 0 to 40 m/sec. The unit will measure up to 65 m/sec.

*10-meter Wind Speed* (Vector value in m/sec)=5-minute average of 3-second samples

*10-meter Wind Direction* in degrees=5-minute average of 3-second samples

*10-meter Wind Direction*-*Standard Deviation*

*10-meter Wind Speed-Standard Deviation*

*10-meter Wind Speed*= Peak three-second wind gust (m/sec)

20-ft Wind Speed = 5-minute average of 3-second samples

20-ft anemometer is an R.M. Young 03101 Wind Sentry

2-meter Wind Speed = 5-minute average of 3-second samples

2-meter anemometer is an R.M. Young 03101 Wind Sentry
